# Case report: paraneoplastic cerebellar degeneration associated with anti-Yo antibody successfully treated with ofatumumab

**DOI:** 10.3389/fimmu.2024.1476397

**Published:** 2024-12-16

**Authors:** Jingjing Dou, Xiaotong Yue, Haitao Ren, Chunjuan Wang, Shougang Guo

**Affiliations:** ^1^ Department of Neurology, Shandong Provincial Hospital, Shandong University, Jinan, Shandong, China; ^2^ Department of Neurology, Peking Union Medical College Hospital, Chinese Academy of Medical Sciences, Peking Union Medical College, Beijing, China; ^3^ Department of Neurology, Shandong Provincial Hospital Affiliated to Shandong First Medical University, Jinan, Shandong, China

**Keywords:** paraneoplastic cerebellar degeneration, anti-Yo antibody, CDR2, CDR2L, ofatumumab, CD20

## Abstract

Paraneoplastic cerebellar degeneration (PCD) with anti-Yo antibodies represents a rare immune-mediated paraneoplastic neurological syndrome. Its diagnosis and management remain clinically challenging. Here, we present a case of PCD with confirmed anti-Yo antibodies, validated through anti-cerebellar degeneration protein 2 (CDR2) and anti-CDR2-like antibodies detection, which demonstrated a favorable response to ofatumumab therapy. The patient initially manifested with dizziness, nystagmus, dysarthria, and ataxia. Initial testing revealed weakly positive anti-Yo antibodies, accompanied by positive serum tissue-based assay result for cerebellum. Following one course of intravenous immunoglobulin and methylprednisolone pulse therapy, improvement of the patient’s dizziness was observed. Oral prednisone was prescribed for maintenance therapy. However, after discharge, the patient experienced progressive deterioration of symptoms, including worsening dizziness, dysarthria, and limb ataxia. Upon readmission to our hospital, further immunological testing confirmed the presence of anti-CDR2 and anti-CDR2-like antibodies. When a second course of methylprednisolone pulse therapy proved ineffective, treatment was switched to ofatumumab. After two doses, the patient achieved partial symptomatic relief.

## 1 Introduction

Paraneoplastic cerebellar degeneration (PCD) is a rare immune-mediated disorder with a strong association with underlying malignancies. The diagnosis primarily relies on the detection of highly specific anti-neuronal antibodies in serum and/or cerebrospinal fluid (CSF), including anti-Yo, anti-Hu, anti-Tr, anti-Ri, and anti-Ma2 antibodies. Among these, anti-Yo-mediated autoimmunity represents a particularly aggressive form of paraneoplastic neurological syndrome, predominantly affecting female patients with gynecologic or breast adenocarcinomas. Anti-Yo targets two antigens, cerebellar degeneration-related protein 2 (CDR2) and CDR2-like (CDR2L). Both proteins belong to the cerebellar degeneration-related protein family and are predominantly located in the cytoplasm and proximal dendrites of Purkinje cells. Historically, CDR2 was considered the sole Yo antigen; however, recent evidence has revealed that CDR2L, despite sharing 44.7% sequence identity with CDR2, represents not only another major antigen but may actually be the only Yo antigen in Yo-mediated autoimmunity (1). Conventional commercial assays for anti-Yo antibody detection, which use CDR2 as the sole antigen, demonstrate limited specificity for PCD diagnosis. The incorporation of CDR2L testing has significantly enhanced diagnostic accuracy (2). However, evidence-based treatment protocols for PCD remain limited, resulting in largely empirical therapeutic approaches. First-line treatments typically comprise glucocorticoids, plasma exchange, or intravenous immunoglobulin (IVIG). More recently, targeted therapies such as rituximab (RTX) have been introduced for preventing PCD relapse (3). In this report, we present a case of PCD in a patient with anti-Yo antibodies, confirmed through both anti-CDR2 and anti-CDR2L antibodies detection, who demonstrated a favorable response to ofatumumab (OFA) after showing insufficient improvement with conventional methylprednisolone and IVIG therapy.

## 2 Case presentation

A 63-year-old female with a history of hypertension and coronary heart disease initially presented with progressive neurological symptoms over one month, beginning with intermittent dizziness and vomiting, later developing slurred speech and impaired motor coordination. Notably, she had experienced herpes zoster affecting the right frontal region two weeks prior to symptom onset. Upon admission, the neurological examination revealed bilateral horizontal and vertical nystagmus, slurred speech, and a broad-based gait. Finger-to-nose and heel-to-shin tests showed dysmetria. Hyporeflexia was noted in the upper limbs, while the tendon reflexes in the lower extremities were preserved. Increased muscle tone was noted in both upper extremities. No sensory deficits were observed. Babinski and Chaddock signs were positive bilaterally. The patient was unable to complete the Romberg sign test, further indicating balance and coordination difficulties. The cognitive assessment revealed a Mini Mental State Examination (MMSE) score of 27/30 (adjusted for primary school education level), and a Montreal Cognitive Assessment (MoCA) score of 25/30. Additionally, the patient’s international cooperative ataxia rating scale (ICARS) score was 67/100, reflecting the severity of her ataxia symptoms.

**Figure 1:**
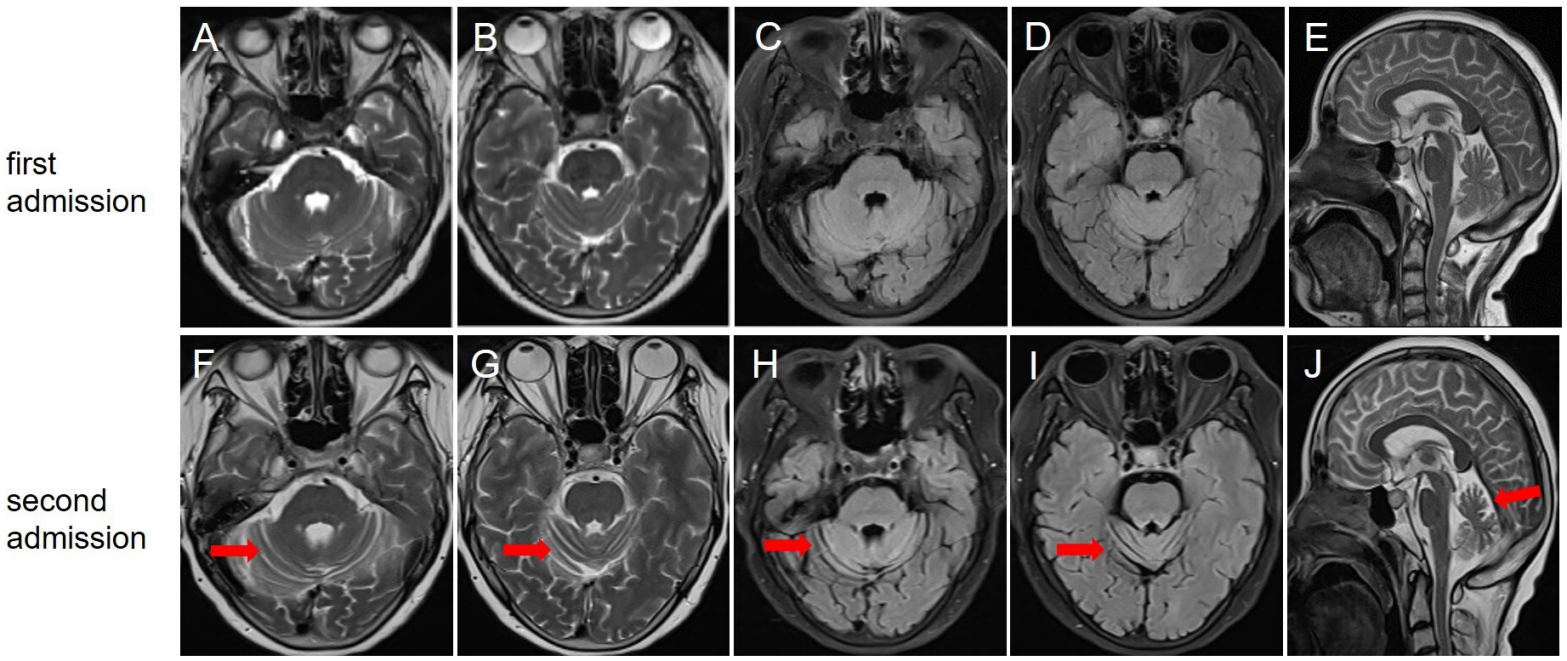
Brain magnetic resonance imaging at the first **(A–E)** and second **(F–J)** admission. T2-weighted **(A, B)**, fluid-attenuated inversion-recovery **(C, D)**, and sagittal view **(E)** showed no significant cerebellar atrophy; **(F, G)** T2-weighted showed that the bilateral cerebellar hemispherical sulci were significantly wider and deeper than before; **(H, I)** Fluid-attenuated inversion-recovery sequence showed cerebellar atrophy; **(J)** Sagittal view indicated cerebellar atrophy.

**Figure 2:**
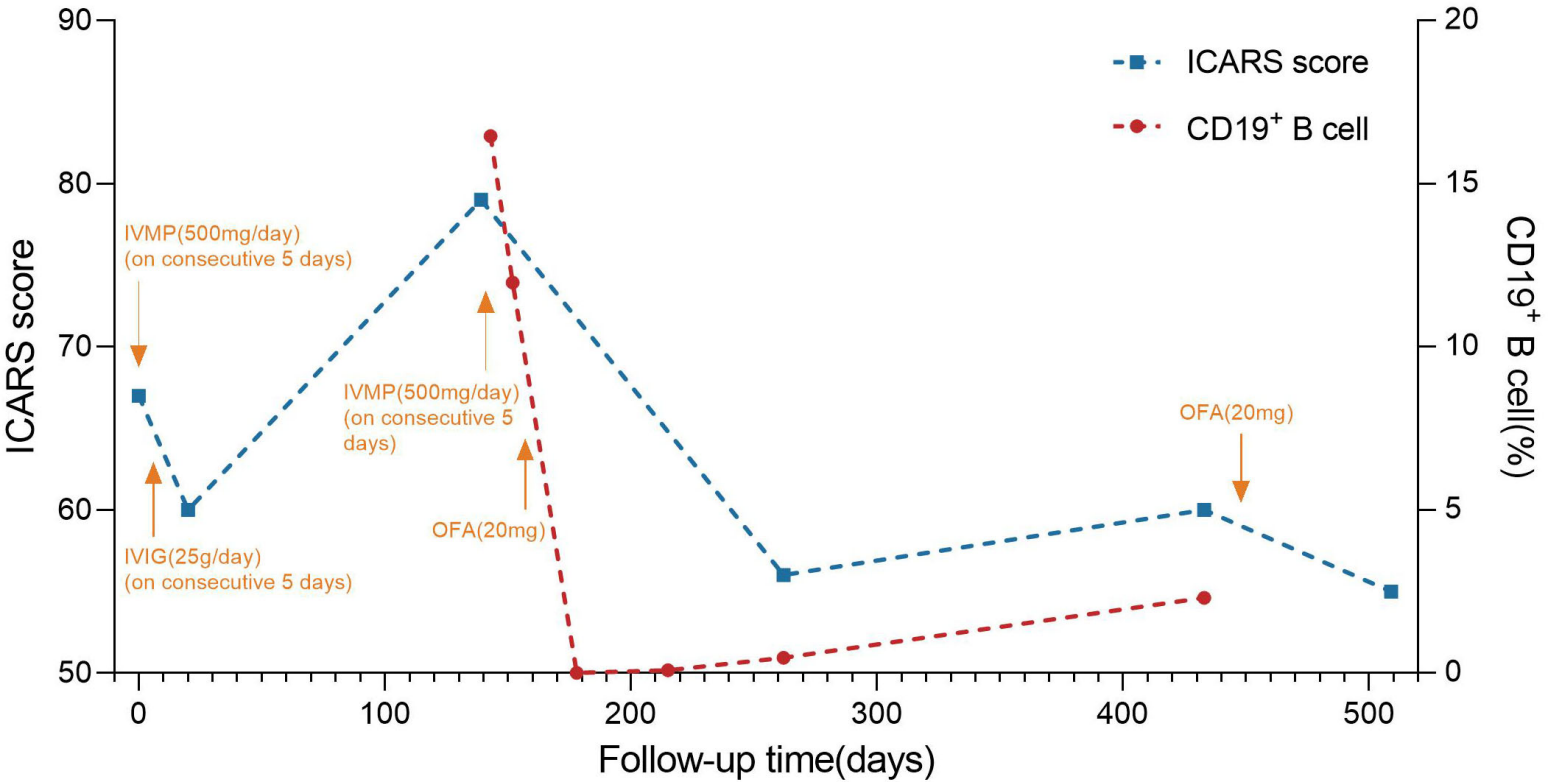
Timeline of disease course and different treatment regimes. The x-axis indicates the number of days after the first admission. The left y-axis indicates international cooperative ataxia rating scale (ICRAS) score. The right y-axis indicates the percentage of CD19^+^ B cell. ICRAS score is used to describe and quantitatively assess cerebellar ataxia symptoms, ranging from 0 to 100, with higher scores indicating more severe coordination dysfunction. IVIG: 0.4 g/kg intravenous immunoglobulins daily for 5 days; IVMP: intravenous methylprednisolone 500mg for 5 consecutive days, and gradually reduced to OP. ICARS score fluctuated throughout the entire immunotherapy process. The patient was given IVMP and IVIG therapy after the first admission, and the patient’s ICARS score decreased from 67 to 60 at the first discharge. After discharge, the patient’s symptoms gradually worsened, and the ICARS score increased to 79 on the second admission. After the second cycle of IVMP treatment, OFA was subcutaneously injected (20 mg). On day 21 after the first injection, the percentage of CD19^+^ B cells decreased to 0%. 9 months after the first injection, the percentage of CD19^+^ B cells rose to 2.3%. A second subcutaneous OFA injection of 20mg was administered. ICARS score was 55/100 at 2 months after the second injection. IVMP, intravenous methylprednisolone; IVIG, intravenous immunoglobulin; OP, oral prednisone; OFA, ofatumumab.

**Figure 3:**
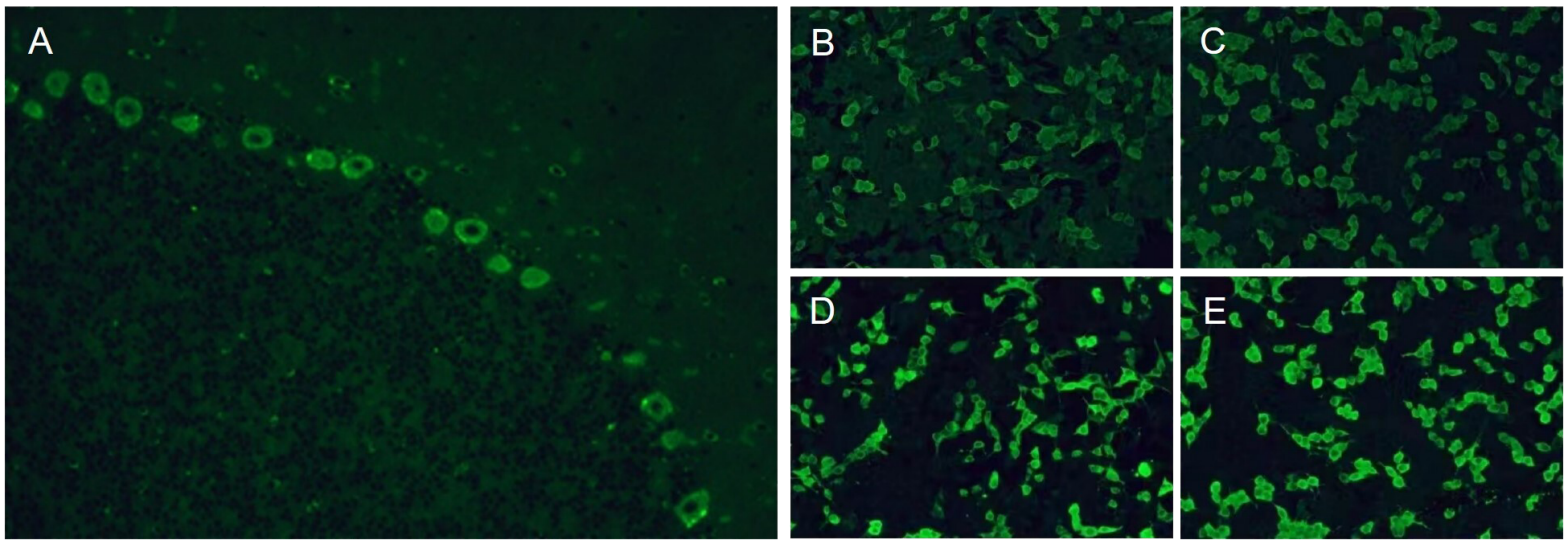
Tissue-based assay test showed positive immunofluorescence of cerebellar Purkinje cells **(A)**. Cell-based assay test showed positive immunofluorescence of serum **(B)** and cerebrospinal fluid **(D)** by reaction with the CDR2 antigen. Cell-based assay test showed positive immunofluorescence of serum **(C)** and cerebrospinal fluid **(E)** by reaction with the CDR2L antigen.

After her admission, extensive screening of infections (human immunodeficiency virus antigen/antibody, treponema pallidum antibody, cytomegalovirus DNA, Epstein-Barr virus DNA, herpes simplex virus DNA, and varicella zoster virus DNA), metabolic disorders (homocysteine, folate and vitamin B12 levels), systemic autoimmune diseases (antinuclear antibodies, anti-Sm, anti-dsDNA, anti-SSA and anti-SSB antibodies, thyroglobulin and thyroid peroxidase antibodies) and serum tumor markers yielded negative results. The lumbar puncture revealed normal pressure (125mmH_2_O), pleocytosis with a white blood cell count of 25×10^6^/L (mononuclear cell count of 24×10^6^/L), and an elevated protein level of 1.41 g/L. Brain magnetic resonance imaging (MRI) results were also unremarkable (**Figure 1**). The routine tests performed by cell-based assay for an encephalitis antibody panel including anti-mGluR1 antibodies of serum and CSF (Simcere, China) and a demyelinating antibody panel including anti-aquaporin-4 antibodies, anti-glial fibrillary acidic protein antibodies, anti-myelin basic protein antibodies and anti-myelin oligodendrocyte glycoprotein antibodies of serum (Simcere, China) were all negative. Anti-Yo antibody was weakly positive. Serum tissue-based assay test for the cerebellum (Simcere, China) was positive. Initial treatment with one cycle of IVIG and methylprednisolone pulse therapy yielded partial improvement, with ICARS score decreasing to 60/100. Oral prednisone was prescribed for maintenance therapy. However, when the dose was reduced to 0.65g/kg, the patient experienced significant clinical deterioration including worsening dizziness, slurred speech and limb ataxia, developing additional symptoms including dysphagia, choking on liquids, repetitive language, agitation, and insomnia. Five months after her last discharge, she was referred back to our hospital. Neurological physical examination revealed poor mental state, hypopharyngeal reflexes, muscle weakness, hypotonia, and severe ataxia in both upper and lower limbs. No sensory deficits were observed. Her ICARS score had increased to 79/100 (**Figure 2**). A repeated lumbar puncture showed normal pressure (80mmH_2_O), pleocytosis with a white blood cell count of 4×10^6^/L (mononuclear cell count of 4×10^6^/L), and an elevated protein level of 0.89g/L. The electroencephalogram demonstrated a slow background rhythm. The brain MRI showed cerebellar atrophy (**Figure 1**). Additionally, the ultrasound examination revealed lymphadenopathy and left-sided breast nodules (breast imaging reporting and data system category 3), prompting a whole-body positron emission tomography/computed tomography scan, which did not identify any tumors. On a fixed rat cerebellum section, the cytoplasm, dendrites, and axons of Purkinje cells were intensely labeled by the patient’s serum and CSF (**Figure 3A**). Further antibody identification and typing confirmed the presence of both anti-CDR2 (**Figures 3B, D**) and anti-CDR2L antibodies (**Figures 3C, E**).

The patient received another cycle of methylprednisolone pulse therapy, but her symptoms did not exhibit significant improvement. We then considered the administration of targeted drugs to alleviate symptoms and prevent future relapses. After carefully excluding the contraindications and obtaining informed consent, subcutaneous OFA (20mg) was prescribed. The peripheral CD19^+^ B cell count decreased to 0 cells/ul, leading to partial symptom relief. At the 4-month follow-up, she was walker-dependent but capable of standing independently. The ICARS score decreased to 50/100. By the 9-month follow-up, she experienced symptom recurrence including worsening dizziness and walking instability, with an ICARS score of 60/100. Meanwhile, the CD19^+^% rose to 2.3% (**Figure 2**). After the second time of positron emission tomography/computed tomography scan, which revealed no evidence of tumors, a second subcutaneous injection of OFA (20mg) was administered. The patient achieved gradual improvement, with an ICARS score of 55/100 two months after the second injection. Notably, no infections were observed during the entire follow-up period.

## 3 Discussion

In 1976, Trotter’s team first identified the existence of anti-cerebellar Purkinje cell antibodies in the serum of Hodgkin’s lymphoma patients with cerebellar degeneration. Subsequently, Greenlee and Brashear described the association between PCD and anti-Yo antibodies in 1983 (4). Anti-Yo-mediated PCD typically manifests with characteristic cerebellar dysfunctions, including cerebellar ataxia, dysarthria, and spontaneous nystagmus. Anti-Yo antibodies bind to the CDR2 and CDR2L which are present in the cytoplasm and proximal dendrites of Purkinje cells, resulting in the loss of Purkinje cells and cerebellar dysfunction (5, 6). Early changes in PCD encompass mild perivascular cuffing by lymphocytes, microglial activation and the infiltration of the cerebellar Purkinje layer by CD8 lymphocytes (7). Despite sharing a 44.7% sequence identity with CDR2, CDR2L fulfills distinct roles. CDR2L interacts with cytosolic ribosomes through rpS6 and appears to function in protein synthesis, while CDR2 associates with nuclear speckle proteins and appears to be involved in mRNA maturation (8). Although commercial line assays are available, concerns have been raised regarding their diagnostic accuracy. In the diagnosis of PCD, there is an approximate 70% false positivity rate when using commercial assays alone (2). A retrospective study, approved by the Mayo Clinic Institutional Review Board, indicated that CDR2L in the line blot format improved sensitivity in CSF and was optimized for specificity in serum when combined with CDR2, and pairing CDR2 and CDR2L may help overcome some interpretative issues, particularly for low positive results in serum (9). Another study developed an in-house cell-based assay to test for anti-CDR2L antibodies and used it to screen sera from 48 patients with confirmed anti-Yo-associated PCD alongside a control group. The results indicated that the combination of anti-CDR2 and anti-CDR2L yielded the most reliable test results (10). Therefore, the incorporation of CDR2L into commercially available line immunoassays for Yo antibody detection is recommended (2). In our case, western blotting and the construction of the CDR2 and CDR2L antigenic sites were instrumental in clarifying the patient’s diagnosis, thereby guiding the selection of an appropriate immunotherapy regimen. Additionally, given the association of anti-Yo antibodies with carcinomas such as breast and ovarian cancer, closely monitoring for tumor occurrence during follow-up is imperative.

As PCD has the potential to cause neuronal destruction, rapid diagnosis and treatment are crucial to prevent irreversible neurological damage. Early treatment is correlated with a more favorable prognosis, highlighting the therapeutic principle of “Time is Cerebellum” (11). The likelihood of clinical improvement in patients with longstanding symptoms and extensive neuronal loss is poor, therefore, once the diagnosis of PCD is confirmed, the immunotherapy should begin as soon as possible. The anti-CDR2/CDR2L antibody-associated PCD often develops carcinomas (breast or ovarian) in women. Tumor therapies are the first choice for PCD with neoplasms. Besides, various immunotherapies are considered, including glucocorticoids, IVIG, immunosuppressants, plasma exchange, RTX, and so on. Concerning PCD associated with anti-Yo antibodies, glucocorticoids seem ineffective, whereas plasma exchange and RTX may offer some benefits (5). Notably, a large number of antibody-secreting cells and CD8^+^T cells can be seen in ovarian tumors of PCD patients with anti-Yo antibodies, and 80% of tumors have a high plasma cell density (12), suggesting that humoral immunity may play a significant role in the pathogenesis of anti-Yo antibodies PCD, so the application of B cell depletion agents could be an effective treatment option. Studies have shown that clinical improvement has been observed in patients with PCD treated with RTX. RTX, a chimeric anti-CD20 monoclonal antibody, is widely used in the treatment of autoimmune diseases but is limited by side effects, such as acute allergic reactions and increased infection risk. OFA represents a significant advancement over traditional anti-CD20 therapies such as RTX, offering several advantages. It is a novel, fully humanized anti-CD20 monoclonal antibody, which can induce the lysis and elimination of pathogenic B cells and reduce the re-production of autoimmune antibodies through the combination of two unique epitopes on CD20-expressing B cells (13). By binding to both the smaller and larger loops of CD20 receptors, OFA achieves higher B cell lysis efficiency than RTX (14). Moreover, OFA can nearly deplete circulating B cells while relatively preserving the rapid recovery of marginal zone B cells and follicular B cells, thereby better maintaining the immune response and hardly affecting the number of naive T cells (15). In terms of safety, previous literature has reported that the incidence of adverse events such as upper respiratory tract infection and injection-related reactions was lower in patients treated with OFA (16, 17). In addition, OFA can be self-administered at home, which is more convenient and economical for the patients. In our case, the selection of OFA was based on multiple considerations. The patient had a long disease course, malnutrition, and a compromised immune system following high-dose methylprednisolone pulse therapy which increases infection susceptibility. And the patient did not consent to plasma exchange therapy. Considering OFA’s characteristics and the patient’s condition, we chose OFA over RTX. Despite being an off-label drug, OFA’s mechanism suggests its potential for treating PCD. In our case, the observed clinical improvement and rapid B cell depletion following OFA administration suggest its potential efficacy in anti-Yo PCD treatment.

This is the first report to investigate the potential of OFA for the treatment of PCD associated with anti-Yo antibodies. We present a PCD case with anti-Yo antibodies, featuring cerebellar atrophy on brain MRI and refractoriness to a second course of methylprednisolone pulse therapy. Clinical improvement and a rapid decline in CD19^+^ B cell count were observed after the administration of OFA. This pioneering report demonstrates the potential utility of OFA in treating anti-Yo antibody-associated PCD. Our findings suggest that OFA may represent a viable therapeutic option, particularly in cases refractory to conventional treatments. However, more large-scale observational studies are needed to further verify the efficacy and safety of OFA for the treatment of PCD.

## Data Availability

The original contributions presented in the study are included in the article/supplementary material. Further inquiries can be directed to the corresponding authors.
